# Diffusion of effects of the ASSIST school‐based smoking prevention intervention to non‐participating family members: a secondary analysis of a randomized controlled trial

**DOI:** 10.1111/add.14862

**Published:** 2019-12-04

**Authors:** James White, Jo Holliday, Rhian Daniel, Rona Campbell, Laurence Moore

**Affiliations:** ^1^ Centre for Trials Research Cardiff University Neuadd Meirionnydd Cardiff UK; ^2^ Nuffield Department of Population Health, Big Data Institute University of Oxford Oxford UK; ^3^ School of Medicine Cardiff University Neuadd Meirionnydd Cardiff UK; ^4^ Population Health Sciences, Bristol Medical School, University of Bristol Bristol UK; ^5^ MRC/CSO Social and Public Health Sciences Unit University of Glasgow Glasgow UK

**Keywords:** Diffusion, family, peers, prevention, smoking, spillover

## Abstract

**Aims:**

To investigate whether effects of the ASSIST (A Stop Smoking In Schools Trial) school‐based smoking prevention intervention diffused from students to the people they lived with.

**Design:**

Secondary analysis of a cluster‐randomized control trial (cRCT).

**Setting:**

England and Wales.

**Participants:**

A total of 10 730 students aged 12–13 years in 59 schools assigned using stratified block randomization to the control (29 schools, 5372 students) or intervention (30 schools, 5358 students) condition.

**Intervention and comparator:**

The ASSIST intervention involves 2 days of off‐site training of influential students to encourage their peers not to smoke during a 10‐week period. The control group continued with their usual education.

**Measurements:**

The outcomes were the proportion of students who self‐reported living with a smoker and the smoking status of each resident family member/caregiver. Follow‐up assessments were immediately after the intervention and at 1 and 2 years post‐intervention.

**Findings:**

The odds ratio (OR) for living with a smoker in the intervention compared with the control groups was 0.86 [95% confidence interval (CI) = 0.72, 1.03] immediately after the intervention, OR = 0.84 (95% CI = 0.72, 0.97) at a 1‐year follow‐up and OR = 0.86 (95% CI = 0.75, 0.99) at 2‐year follow‐up. In a three‐tier multi‐level model with data from all three follow‐ups, student‐reported smoking by fathers (OR = 0.90, 95% CI = 0.80, 1.00), brothers (OR = 0.78, 95% CI = 0.67, 0.92) and sisters (OR = 0.80, 95% CI = 0.69, 0.92) was lower in the intervention compared with control group. Subgroup analyses by baseline smoking status suggested that these effects were more consistent with prevention of uptake than prompting cessation.

**Conclusions:**

A Stop Smoking In Schools Trial (ASSIST) school‐based smoking prevention intervention may have reduced the prevalence of smoking in people who lived with ASSIST‐trained students. This indirect transmission is consistent with the predictions of diffusion of innovations theory which underpins the design of ASSIST.

## Introduction

A number of observational studies have found a concordance in smoking initiation, maintenance and cessation among peers 1, 2, 3. In the Framingham Heart Study, social network data collected during a 29‐year period showed that smoking cessation by a spouse decreased a person's chances of smoking by 67%, a sibling by 25% and a friend by 36% 1. There has, however, been less examination on whether intervention effects are transmitted. In the PROmoting School‐community‐university Partnerships to Enhance Resilience (PROSPER) study, friends of participants who received the Strengthening Families Program for Youth 10–14 (SFP) but were unexposed themselves were less likely to get drunk and use cigarettes at a 3‐year follow‐up if they had three or more friends attending the SFP compared with those with no friends attending the SFP 4. However, as neither receipt of SFP nor friendships were randomly assigned, this association may reflect pre‐existing differences in the social networks of families and students in risk factors for student drunkenness and cigarette use.

The ASSIST (A Stop Smoking in Schools Trial) is a school‐based smoking prevention intervention, found to be effective in reducing the prevalence of weekly smoking in students aged 12–13 years 5. In ASSIST, influential students are identified and trained to diffuse non‐smoking information and norms, principally through conversations with their friends. The intervention is delivered in many areas of the United Kingdom, with anecdotal reports from the team that trains ASSIST intervention delivery staff that students have conversations with their family about smoking, suggesting that there may be potential spill‐over effects. In the cluster‐randomized controlled trial (cRCT) of the ASSIST intervention, student reports on the smoking status of family/care‐givers were collected, providing an unusual opportunity to explore whether intervention effects are diffused beyond the original intended group (students) to family members/care‐givers. We conducted exploratory analysis to test the hypothesis that there will be a reduced prevalence of smoking in the families/care‐givers of students who attended an ASSIST intervention school compared to those in control schools. In subgroup analyses by families/care‐givers’ baseline smoking status we explored potential effects on both smoking uptake and cessation.

## Methods

### Design

ASSIST used a two‐arm cRCT design and was conducted in schools in the West of England and Southeast Wales. In 2001, 223 secondary schools were invited to participate. One hundred and twenty‐seven schools expressed an interest, were visited, and 113 agreed to participate. Sixty‐six schools were randomly sampled from these 113 with stratification by country, type of school (independent or state), mixed‐ or single‐sex, English‐ or Welsh‐speaking, size (< 200 or ≥ 200 students) and level of entitlement to free school meals (above or below the median entitlement of 19%). Of these 66 schools, 59 signed an agreement to be randomized. The Multi‐Centre Research Ethics Committee for Wales reviewed the trial protocol and judged it as meeting ethically acceptable standards. The current analysis was not proposed in the study protocol and uses data gathered at baseline (September 2001–February 2002), immediately after the intervention (January 2002–May 2002) and at 1 year (Nov 2002–May 2003) and 2‐year follow‐ups (November 2003–May 2004) 6. The paper adheres to the Consolidated Standards of Reporting Trials (CONSORT) guidelines on the reporting of cRCTs 7.

### Procedures

Stratified block randomization was used with strata defined by the same criteria as random sampling. Written consent was obtained from parents on an opt‐out basis and students provided written assent. Full details of the study design and data collection methods can be found elsewhere (ISRCTN 55572965) 6.

### Intervention

ASSIST is an informal peer‐led smoking prevention intervention based on diffusion of innovations theory (see Supporting information, Table S1 for a full description) 8. It aims to diffuse and sustain non‐smoking norms via secondary school students’ social networks in UK year 8 (aged 12–13). In intervention schools, 18.7% of students were trained to be peer supporters (range across schools = 14.8–24.6%).

### Measures and outcomes

At baseline, students were asked to complete a questionnaire which included questions on their age, sex, the family affluence scale 9 and smoking behaviour. Students at 12 intervention and 12 control schools provided a saliva sample for cotinine analysis at 2‐year follow‐up to minimize reporting bias 10. At baseline and each of the three post‐intervention data collections students were also asked: ‘Does anyone who lives in your house smoke tobacco, e.g. cigarettes, cigars or pipes? Please don't include yourself’. This was coded into a binary variable of ‘any smoker’ or not. Those who lived with a smoker were asked to write who the smokers were. Responses were coded into the outcomes of residence with a smoking: mother, father, brother, sister, grandmother and grandfather. Preliminary analysis showed that few students lived with other family members/care‐givers who smoked. Only 1.9% had an aunt who smoked, 2.1% a smoking uncle, 1.3% a boyfriend of a parent who smoked and 0.3% a girlfriend of a parent who smoked. Analysis was not conducted for these family members/care‐givers, but they were included in analysis of the ‘any smoker’ group. Analyses were run for each of seven outcomes—student‐reported smoking of a mother, father, brother, sister, grandmother, grandfather and any smoker.

### Statistical methods

Three multi‐level logistic regression models (students nested within schools) were fitted with the outcome being smoking prevalence, separately for the three follow‐up occasions: immediately after the intervention and at the 1‐ and 2‐year follow‐ups. As predictors, each of these models included the five school‐level stratifying variables, the family affluence score, family car ownership and the respective family/care‐giver's smoking behaviour at baseline. To allow individuals with missing measures at follow‐ups to be included in the analysis and reduce bias because of loss to follow‐up 11, we also carried out analyses with a three‐level model using data from all follow‐up periods together; schools were at level 3, students at level 2 and follow‐up measurements at level 1. Model parameters were estimated with first‐order penalized quasi‐likelihood within MLwin (version 3.02) using the *runmlwin* command in Stata (version 15.0).

We conducted multiple subgroup analyses. To examine the effects of the intervention on uptake and cessation we conducted separate analyses according to family/care‐givers’ baseline smoking status. This analysis was repeated after imputing missing data as if the family members’ unknown follow‐up smoking status had not changed from its observed value at baseline. To test the hypothesis that effects may only occur in family/care‐givers who lived with peer supporters (who are trained to diffuse and are therefore exposed to more non‐smoking messages than non‐peer supporter students), we re‐ran analyses after excluding nominated peer supporters from the control and intervention condition. To examine whether smoking cessation in students explained any indirect effect of ASSIST, we re‐ran analyses after excluding students who smoked at baseline. As analyses were exploratory in nature, we did not adjust for multiple comparisons 12. All analysis was by intention‐to‐treat.

## Results

Supporting information, Figure S1 shows the trial profile. Two schools withdrew after randomization and were replaced by one from the same strata of interested schools. Of the 11 043 potentially eligible students in the 59 participating schools, 313 (3%) were withdrawn by their parents or carers before collection of data at baseline. Twenty students were excluded, as they indicated that they did not live with a resident who smoked, but then named a relative. At every data collection point more than 80% of eligible students provided information on whether the people they lived with smoked. The final analytical sample included 10 730 students (59 schools), 5372 (across 29 schools) in the control arm and 5358 (30 schools) in the intervention arm.

The proportion of students that lived with a smoker decreased from 54.2% (5460 of 10 066) at baseline to 49.7% (4531 of 9123) at the 2‐year follow‐up. At baseline, mothers (31.7%) and fathers (30.9%) were most common family member to smoke in the household (Table 1). The proportion of students that lived with a smoker was lower in the intervention than control group at baseline (51.2 versus 57.4%) and all three follow‐up assessments (immediately after the intervention: 48.9 versus 54.7%; 1 year: 48.4 versus 54.4%; 2 years: 46.7 versus 52.8%). The odds ratio (OR) for living with a smoker in the intervention compared to control group was 0.86 [95% confidence interval (CI) = 0.72, 1.03] immediately after the intervention, OR = 0.84 (95% CI = 0.72, 0.97) at 1‐year follow‐up and OR = 0.86 (95% CI = 0.75, 0.99) at 2‐year follow‐up (Table 2). In the three‐tier multi‐level model, with data from all three follow‐ups, the odds of students living with a smoking fathers (OR = 0.90, 95% CI = 0.80, 1.00), brothers (OR = 0.78, 95% CI = 0.67, 0.92) and sisters (OR = 0.80, 95% CI = 0.69, 0.92) were lower in the intervention than control arm (Supporting information, Fig. S1).

**Table 1:** Baseline characteristics of schools, students and people who live with students by experimental group.

	Control	Intervention
Schools		
Total (*N* = 59)	29 (49%)	30 (51%)
Independent	1 (3%)	2 (7%)
State	28 (97%)	28 (93%)
Welsh language	2 (7%)	1 (3%)
English language	27 (93%)	29 (97%)
Free school meals		
> 19% student entitlement	12 (41%)	14 (47%)
≤ 19% student entitlement	17 (59%)	16 (53%)
Size		
≥ 200 students	13 (45%)	13 (43%)
< 200 students	16 (55%)	17 (57%)
Location		
England	17 (59%)	15 (50%)
Wales	12 (41%)	15 (50%)
Students		
Total (*N* = 10 710)	5362 (50.1)	5348 (49.9)
Smoking behaviour		
Never smoker	2716/5077 (54.9)	2875/5077 (56.6)
Occasional, experimental or ex‐smoker	1909/5077 (38.6%)	1959/5077 (38.6%)
Weekly smoker	327/5077 (6.6%)	243/5077 (4.7%)
Boys	2752/5362 (51.3%)	2739/5348 (51.2)
Family affluence score		
0–2	1274/4765 (26.7%)	1144/4984 (23.0%)
3–4	2596/4765 (54.5%)	2775/4984 (55.7%)
5–6	895/4765 (18.8%)	1065/4984 (21.4%)
Family vehicle ownership		
No family car or van	354/4808 (7.4%)	295/5008 (5.9%)
One family car or van	2088/4808 (43.4%)	1849/5008 (36.9%)
Two or more cars or vans	2366/4808 (49.2%)	2864/5008 (57.2%)
Smokers who live with the student		
Mother	1670/4965 (33.6%)	1517/5101 (29.7%)
Father	1638/4965 (32.9%)	1470/5101 (28.8%)
Brother	380/4965 (7.7%)	359/5101 (7.0%)
Sister	335/4965 (6.7%)	322/5101 (6.3%)
Grandmother	266/4965 (5.4%)	240/5101 (4.7%)
Grandfather	252/4965 (5.1%)	218/5101 (4.3%)
Boyfriend of parent	60/4965 (1.2%)	70/5101 (1.4%)
Girlfriend of parent	13/4965 (0.3%)	14/5101 (0.3%)
Living with a smoker	2848/4965 (57.4%)	2612/5101 (51.2%)

Data are *N* (%) or *n*/*N* (%) unless otherwise specified.

**Table 2:** Odds ratios for the intervention effect on the smoking status of people who live with students at every follow‐up.

	Immediately after the intervention		1‐year follow‐up		2‐year follow‐up	
	*n*	OR (95% CI)	*n*	OR (95% CI)	*n*	OR (95% CI)
Person with whom student lives^a^						
Mother	9398	1.22 (0.96, 1.54)	8846	0.80 (0.68, 0.94)	8410	0.81 (0.68, 0.97)
Father	9642	0.95 (0.80, 1.13)	8846	0.89 (0.77, 1.02)	8410	0.92 (0.78, 1.07)
Brother	8935	0.76 (0.60, 0.97)	8846	0.72 (0.60, 0.86)	8410	0.88 (0.73, 1.04)
Sister	8935	0.69 (0.56, 0.86)	8846	0.80 (0.66, 0.96)	8410	0.91 (0.76, 1.09)
Grandmother	8935	1.03 (0.81, 1.32)	8846	1.00 (0.78, 1.28)	8410	0.85 (0.64, 1.13)
Grandfather	8935	0.95 (0.76, 1.20)	8846	1.06 (0.78, 1.44)	8410	0.88 (0.66, 1.17)
Living with a smoker	8935	0.86 (0.72, 1.03)	8846	0.84 (0.72, 0.97)	8410	0.86 (0.75, 0.99)

a Adjusted for baseline smoking status of resident, student gender, family affluence score (0–2, 3–4, 4–6), family vehicle ownership (no family car or van, one family car or van, two family cars or vans) and stratification variables (country: England or Wales; type of school: independent or state; mixed‐sex or single‐sex; English‐ or Welsh‐speaking; size of school year group: < 200, ≥ 200; % students entitled to free school meals: ≤ 19%, > 19%). OR = odds ratio; CI = confidence interval.

In subgroup analyses, the odds of smoking uptake were lower for fathers, brothers and sisters immediately after the intervention and the 1‐year follow‐up in the intervention than control arm (Supporting information, Table S2 **)**. There was little evidence of a beneficial effect on smoking cessation. Analysis into smoking uptake and cessation where missing data was imputed with baseline smoking status (Supporting information, Table S3 **)**, which removed peer supporters (Supporting information, Fig. S2 **)** or students who smoked at baseline from analyses (Supporting information, Fig. S3 **)**, a had little impact on estimates.

## Discussion

These exploratory analyses suggest that a school‐based smoking prevention intervention may have reduced the prevalence of smoking in people who live with students. These residents did not directly receive the intervention. These analyses were not part of the original trial protocol. As such, they require replication in an independent study before informing practise.

### Comparison with existing studies

The transmission of smoking behaviours to siblings that we found replicates the results from the social network analysis in the Framingham study 1, and the indirect effect of the SFP family‐based substance use prevention programme on cigarette use in friends of participants 4. Our analysis has extended the results from these studies by finding evidence of diffusion of an intervention effect from adolescents to family members not directly exposed. Importantly, as ASSIST was an RCT, the potential for a confounding effect of participant selection into an intervention and individual and network‐level differences in risk factors for smoking status was minimized. Although we are aware of diffusion of intervention effects in RCTs evaluating weight loss 13 and bariatric surgery 14, to our knowledge this is first evidence of diffusion of an intervention effect to smoking behaviour, and from adolescent to a parent. The transmission of effects is consistent with the predictions of diffusion of innovations theory 8 on which ASSIST is based.

Among the candidate mechanisms explaining a beneficial effect of ASSIST on family/care‐givers, one hypothesis consistent with the associations observed in the Framingham study is that ASSIST prompted smoking cessation in students which, in turn, influenced family/care‐givers’ smoking status 1. A subgroup analysis excluding students who smoked produced estimates comparable to the main results. This suggests that students did not need to stop smoking to influence other family/care‐givers’ smoking status. Another explanation is that peer supporters carried on their role of passing on messages informally to encourage non‐smoking at home. The subgroup analysis according to baseline family member smoking status suggests that the effect of the intervention on smoking prevalence was more consistent with preventing uptake than promoting cessation. That effects remained similar for all outcomes when peer supporters were excluded from analysis suggests that the spill‐over effects of the ASSIST intervention to those who lived with students occurred across the whole year group, not just among the families of peer supporters.

### Strengths and weaknesses

The strengths of this study are that it is the first to examine the indirect effect of a school‐based intervention to parents, siblings and grandparents. There was some differential loss to follow‐up in the original sample according to student smoking behaviour and family affluence. The adjustment for these variables would have acted to minimize any bias introduced by differential loss to follow‐up, assuming dropout at random, and would not have explained the effects we observed. There were imbalances in the proportion of residents who smoked between arms at baseline. As all analyses adjusted for the baseline smoking status of resident(s), these imbalances do not preclude valid inferences being drawn about the intervention effect 15. Outcomes were all self‐reported, and there could be differential reporting bias between intervention and control arms 16. However, as described elsewhere, no difference was found in ASSIST between students who self‐reported not smoking and had a salivary cotinine concentration greater than 15 ng/ml between groups, suggesting that any bias in student self‐reported smoking was balanced 5. The motivation for conducting these analyses emerged from feedback from the ASSIST implementation team and was hypothesis‐driven; however, interpretation should be cautious, as they were not pre‐registered and require independent confirmatory studies, ideally with more recent data than these data gathered in 2001–04.

## Conclusions

Our findings suggest that a school‐based smoking prevention intervention may have reduced smoking among non‐participating family members/care‐givers. If these findings are replicated, it would suggest that outcomes targeted by an intervention should be collected on those who might be indirectly exposed (e.g. spouses, family members, siblings, friends, co‐workers) to gain a more comprehensive account of potential benefits. It also suggests that greater attention should be paid to network‐level processes which might facilitate diffusion of effects in intervention design.

## Clinical trial registration

Current Controlled Trials ISRCTN 55572965.

## Declaration of interests

L.M., R.C. and J.W. are scientific advisers to Evidence to Impact http://evidencetoimpact.com/, a not‐for‐profit organization that licenses ASSIST. All other authors declare no competing interests.

## Supporting information


**Fig S1** CONSORT flow diagram
**Fig S2** Odds ratios from multilevel model for intervention effect on smoking status in people who live with students
**Fig S3** Odds ratios from a multilevel model for the intervention effect on smoking status after excluding peer supporters
**Fig S4** Odds ratios from a multilevel model for the intervention effect on smoking status after excluding students who smoked at baseline
**Table S1** Stages in the ASSIST intervention
**Table S2** Odds ratios for the intervention effect on the smoking cessation and uptake at every follow‐up
**Table S3** Odds ratios for the intervention effect on the smoking cessation and uptake at every follow‐up with imputed follow‐up data.
