# Supplementary material for: Diffusion of effects of the ASSIST school‐based smoking prevention intervention to non‐participating family members: a secondary analysis of a randomized controlled trial
Source: Addiction. 2019 Dec 4;115(5):986–91. doi: 10.1111/add.14862 (PMC7156286; doi:10.1111/add.14862)
Supplement: Supplementary file 1 — Fig S1 CONSORT flow diagram Fig S2 Odds ratios from multilevel model for intervention effect on smoking status in people who live with students Fig S3 Odds ratios from a multilevel model for the intervention effect on smoking status after excluding peer supporters Fig S4 Odds ratios from a multilevel model for the intervention effect on smoking status after excluding students who smoked at baseline Table S1 Stages in the ASSIST intervention Table S2 Odds ratios for the intervention effect on the smoking cessation and uptake at every follow‐up Table S3 Odds ratios for the intervention effect on the smoking cessation and uptake at every follow‐up with imputed follow‐up data. [file ADD-115-986-s001.docx]

**Supplementary information**

1. Figure S1. CONSORT flow diagram
2. Table S1. Stages in the ASSIST intervention
3. **Figure S2. Odds ratios from multilevel model for intervention effect on smoking status in people who live with students**
4. Figure S3. Odds ratios from a multilevel model for the intervention effect on smoking status after excluding peer supporters
5. Figure S4. Odds ratios from a multilevel model for the intervention effect on smoking status after excluding students who smoked at baseline
6. **Table S2. Odds ratios for the intervention effect on the smoking cessation and uptake at every follow-up**
7. **Table S3. Odds ratios for the intervention effect on the smoking cessation and uptake at every follow-up with imputed follow-up data**

127 visits to schools by research team

223 potentially eligible schools invited

10 students excluded due to illogical responses on residents smoking

5372 eligible students

5074 (95%) participated

5358 eligible students

5187 (97%) participated

4950 (92%) analysed

5562 eligible students in 29 control schools

5481 eligible students in 30 intervention schools

5087 (95%) analysed

10 students excluded due to illogical responses on residents smoking

190 withdrawn by parents

123 withdrawn by parents

7 schools withdrew

6 schools withdrew;

8 ineligible;

47 schools not selected at random

96 schools not interested

59 committed at agreement stage

66 randomized

**Baseline data collection**

5308 eligible students

4509 (85%) analysed

5303 eligible students

4774 (90%) analysed

55 students left study

10 students join study

58 students left study

4 students join study

**After intervention follow-up**

232 students left study

29 moved to intervention school

212 students join study

47 moved from intervention school

158 students left study

6 moved to a control school

140 students join study

11 moved from a control school

**1-year follow-up**

147 students left study

47 moved to a control school

158 students join study

29 moved from a control school

5296 eligible students

4629 (87%) analysed

**2-year follow-up**

5283 eligible students

4484 (85%) analysed

5274 eligible students

4091 (76%) analysed

5306 eligible students

4333 (82%) analysed

239 students left study

11 moved to an intervention school

212 students join study

6 moved from an intervention school

**Figure S1. CONSORT flow diagram**

**Table S1. Stages in the ASSIST intervention**

| **Nomination of peer supporters**   - Students aged 12–13 years (UK Year 8) were asked to identify influential peers using three questions, “Who do you respect in Year 8 at your school?”, “Who are good leaders in sports or other groups activities in Year 8 at your school?”, and “Who do you look up to in Year 8 at your school?” - The 18% of year 8 pupils receiving the most peer nominations were invited to a recruitment meeting. |
| --- |
| **Recruitment of peer supporters**   - A meeting was held with nominees to explain the role of a peer supporter and answer questions. - Trainers made it clear that students who smoked could only be peer supporters if they commit to trying to stop smoking. |
| **Training of peer supporters**   - Training was held off the school site over two days and delivered by a team of external trainers experienced in youth work and health-promotion. - The aims of the training were to: provide information about risks of smoking and benefits of remaining smoke-free; develop communication skills including, listening, cooperation and negotiation, and conflict resolution; enhance students’ confidence, empathy, assertiveness, attitudes to risk-taking, and exploration of personal values. |
| **Intervention period**   - Ten-week peer-led intervention during which peer supporters had informal conversations with their peers about smoking (for example, when travelling to and from school, in breaks, at lunchtime, and after school in their free-time), and logged conversations in a pro-forma diary. - Four school-based follow-up visits with peer supporters and led by ASSIST trainers aimed to provide support and guidance. |
| **Acknowledgment of peer supporters’ contribution**   - All peer supporters were presented with a certificate. - Peer supporters who handed in their diary were presented with a gift certificate. |


| **Variable** | **N** |  | | |  | | | **Odds Ratio (95% CI)** |
| --- | --- | --- | --- | --- | --- | --- | --- | --- |
| **Person who student lives with** ^a^ |  |  | | |  | | |  |
| Mother | 9576 |  | | |  | | | 0.95 (0.81, 1.11) |
| Father | 9642 |  | | |  | | | 0.90 (0.80, 1.00) |
| Brother | 9472 |  | | |  | | | 0.78 (0.67, 0.92) |
| Sister | 9472 |  | | |  | | | 0.80 (0.69, 0.92) |
| Grandmother | 9472 |  | | |  | | | 0.96 (0.77, 1.19) |
| Grandfather | 9472 |  | | |  | | | 0.93 (0.78, 1.12) |
|  |  |  | | |  | | |  |
| **Living with a smoker** | 9472 |  | | |  | | | 0.84 (0.74, 0.95) |
|  |  | 0.25 | 0.50 | 1.00 | | 1.50 | 1.75 |  |
|  |  |  | | |  | | |  |
|  |  | **Favours treatment** | | | **Favours control** | | | |

**Figure S2. Odds ratios from multilevel model for intervention effect on smoking status in people who live with students**

^a^ Adjusted for baseline smoking status of resident, student gender, family affluence score (0-2, 3-4, 4-6), family vehicle ownership (no family car or van, one family car or van, two family cars or vans) and stratification variables (country: England or Wales; type of school: independent or state; mixed-sex or single-sex; English or Welsh speaking; size of school year group: < 200, ≥200; % students entitled to free school meals: ≤19%, >19%).

| **Variable** | **n** |  | | |  | | | **Odds Ratio (95% CI)** |
| --- | --- | --- | --- | --- | --- | --- | --- | --- |
| **Person who student lives with** ^a^ |  |  | | |  | | |  |
| Mother | 7774 |  | | |  | | | 1.00 (0.85, 1.18) |
| Father | 7804 |  | | |  | | | 0.92 (0.81, 1.03) |
| Brother | 7651 |  | | |  | | | 0.79 (0.67, 0.93) |
| Sister | 7651 |  | | |  | | | 0.77 (0.64, 0.91) |
| Grandmother | 7651 |  | | |  | | | 1.00 (0.80, 1.25) |
| Grandfather | 7651 |  | | |  | | | 0.92 (0.76, 1.12) |
|  |  |  | | |  | | |  |
| **Living with a smoker** | 7651 |  | | |  | | | 0.86 (0.75, 0.98) |
|  |  | 0.25 | 0.50 | 1.00 | | 1.50 | 2.00 |  |
|  |  |  | | |  | | |  |
|  |  | **Favours treatment** | | | **Favours control** | | | |

**Figure S3. Odds ratios from a multilevel model for the intervention effect on smoking status after excluding peer supporters**

^a^ Adjusted for baseline smoking status of resident, student gender, family affluence score (0-2, 3-4, 4-6), family vehicle ownership (no family car or van, one family car or van, two family cars or vans) and stratification variables (country: England or Wales; type of school: independent or state; mixed-sex or single-sex; English or Welsh speaking; size of school year group: < 200, ≥200; % students entitled to free school meals: ≤19%, >19%).

| **Variable** | **n** |  | | |  | | | **Odds Ratio (95% CI)** |
| --- | --- | --- | --- | --- | --- | --- | --- | --- |
| **Person who student lives with** ^a^ |  |  | | |  | | |  |
| Mother | 9066 |  | | |  | | | 0.94 (0.81, 1.09) |
| Father | 9122 |  | | |  | | | 0.90 (0.80, 1.01) |
| Brother | 8988 |  | | |  | | | 0.80 (0.70, 0.93) |
| Sister | 8988 |  | | |  | | | 0.86 (0.73, 1.00) |
| Grandmother | 8988 |  | | |  | | | 0.93 (0.77, 1.12) |
| Grandfather | 8988 |  | | |  | | | 0.94 (0.76, 1.16) |
|  |  |  | | |  | | |  |
| **Living with a smoker** | 8988 |  | | |  | | | 0.85 (0.75, 0.96) |
|  |  | 0.25 | 0.50 | 1.00 | | 1.50 | 2.00 |  |
|  |  |  | | |  | | |  |
|  |  | **Favours treatment** | | | **Favours control** | | | |

**Figure S4. Odds ratios from a multilevel model for the intervention effect on smoking status after excluding students who smoked at baseline**

^a^ Adjusted for baseline smoking status of resident, student gender, family affluence score (0-2, 3-4, 4-6), family vehicle ownership (no family car or van, one family car or van, two family cars or vans) and stratification variables (country: England or Wales; type of school: independent or state; mixed-sex or single-sex; English or Welsh speaking; size of school year group: < 200, ≥200; % students entitled to free school meals: ≤19%, >19%).

**Table S2. Odds ratios for the intervention effect on the smoking cessation and uptake at every follow-up**

|  | **Cessation ^a^** | | | | | | **Uptake ^b^** | | | | | | |
| --- | --- | --- | --- | --- | --- | --- | --- | --- | --- | --- | --- | --- | --- |
|  | **Immediately after the intervention** | | **1-year follow-up** | | **2-year follow-up** | | | **Immediately after the intervention** | | **1-year follow-up** | | **2-year follow-up** | |
|  | **n** | **OR (95% CI)** | **n** | **OR (95% CI)** | **n** | **OR (95% CI)** | | **n** | **OR (95% CI)** | **n** | **OR (95% CI)** | **n** | **OR (95% CI)** |
| **Person who student lives with** ^c^ |  |  |  |  |  | | |  |  |  |  |  |  |
| Mother | 2928 | 1.72 (1.33, 2.22) | 2687 | 0.84 (0.67, 1.04) | 2479 | 0.88 (0.70, 1.11) | | 6470 | 0.67 (0.47, 0.95) | 6159 | 0.76 (0.58, 1.00) | 5931 | 0.73 (0.56, 0.95) |
| Father | 2955 | 1.07 (0.87, 1.31) | 2670 | 0.98 (0.81, 1.18) | 2500 | 0.96 (0.76, 1.21) | | 6687 | 0.80 (0.63, 1.03) | 6176 | 0.81 (0.66, 0.98) | 5910 | 0.87 (0.71, 1.07) |
| Brother | 604 | 0.82 (0.55, 1.20) | 601 | 0.59 (0.41, 0.85) | 552 | 0.85 (0.59, 1.23) | | 8331 | 0.73 (0.55, 0.98) | 8245 | 0.76 (0.61, 0.96) | 7858 | 0.88 (0.73, 1.07) |
| Sister | 552 | 0.75 (0.52, 1.09) | 532 | 1.00 (0.69, 1.44) | 493 | 1.00 (0.69, 1.45) | | 8383 | 0.66 (0.50, 0.87) | 8314 | 0.72 (0.58, 0.91) | 7917 | 0.87 (0.70, 1.07) |
| Grandmother | 446 | 1.08 (0.73, 1.60 | 444 | 1.04 (0.69, 1.58) | 414 | 0.91 (0.57, 1.44) | | 8489 | 1.06 (0.75, 1.49) | 8402 | 0.96 (0.69, 1.34) | 7996 | 0.81 (0.55, 1.19) |
| Grandfather | 400 | 1.19 (0.79, 1.81) | 410 | 1.22 (0.77, 1.95) | 369 | 1.03 (0.63, 1.69) | | 8535 | 0.87 (0.65, 1.15) | 8436 | 0.99 (0.67, 1.44) | 8041 | 0.80 (0.54, 1.18) |
|  |  |  |  |  |  |  | |  |  |  |  |  |  |
| **Living with a smoker** | 4706 | 0.92 (0.74, 1.16) | 4662 | 0.85 (0.71, 1.04) | 4347 | 0.87 (0.73, 1.04) | | 4229 | 0.77 (0.60, 1.00) | 4184 | 0.82 (0.67, 1.00) | 4063 | 0.85 (0.70, 1.02) |

^a^ Sample only includes baseline smokers; ^b^ Sample only includes baseline non-smokers; ^c^ Adjusted for baseline smoking status of resident, student gender, family affluence score (0-2, 3-4, 4-6), family vehicle ownership (no family car or van, one family car or van, two family cars or vans) and stratification variables (country: England or Wales; type of school: independent or state; mixed-sex or single-sex; English or Welsh speaking; size of school year group: < 200, ≥200; % students entitled to free school meals: ≤19%, >19%).

**Table S3. Odds ratios for the intervention effect on the smoking cessation and uptake at every follow-up with imputed follow-up data**

|  | **Cessation ^a^** | | | | | | **Uptake ^b^** | | | | | | |
| --- | --- | --- | --- | --- | --- | --- | --- | --- | --- | --- | --- | --- | --- |
|  | **Immediately after the intervention** | | **1-year follow-up** | | **2-year follow-up** | | | **Immediately after the intervention** | | **1-year follow-up** | | **2-year follow-up** | |
|  | **n** | **OR (95% CI)** | **n** | **OR (95% CI)** | **n** | **OR (95% CI)** | | **n** | **OR (95% CI)** | **n** | **OR (95% CI)** | **n** | **OR (95% CI)** |
| **Person who student lives with** ^c^ |  |  |  |  |  | | |  |  |  |  |  |  |
| Mother | 3030 | 1.87 (1.44, 2.42) | 3030 | 0.80 (0.65, 1.00) | 3030 | 0.82 (0.66, 1.02) | | 6612 | 0.64 (0.45, 0.90) | 6612 | 0.78 (0.59, 1.02) | 6612 | 0.77 (0.59, 1.01) |
| Father | 2955 | 1.07 (0.87, 1.31) | 2955 | 0.96 (0.80, 1.16) | 2955 | 0.91 (0.72, 1.15) | | 6687 | 0.80 (0.63, 1.03) | 6687 | 0.84 (0.69, 1.03) | 6687 | 0.93 (0.76, 1.14) |
| Brother | 684 | 0.78 (0.53, 1.14) | 684 | 0.55 (0.38, 0.78) | 684 | 0.78 (0.54, 1.11) | | 8331 | 0.73 (0.55, 0.98) | 8958 | 0.75 (0.56, 1.01) | 8958 | 0.79 (0.63, 0.99) |
| Sister | 617 | 0.73 (0.51, 1.09) | 617 | 0.93 (0.66, 1.33) | 617 | 0.89 (0.63, 1.25) | | 8958 | 0.75 (0.56, 1.01) | 8958 | 0.79 (0.63, 0.99) | 8958 | 0.92 (0.74, 1.13) |
| Grandmother | -^d^ | - | 489 | 0.99 (0.53, 1.13) | 489 | 0.96 (0.62, 1.49) | | 9153 | 1.09 (0.77, 1.54) | 9153 | 0.99 (0.71, 1.39) | 9153 | 0.86 (0.58, 1.26) |
| Grandfather | 451 | 1.23 (0.84, 1.81) | 451 | 1.05 (0.69, 1.58) | 451 | 0.98 (0.65, 1.46) | | 9191 | 0.89 (0.67, 1.19) | 9191 | 1.01 (0.69, 1.48) | 9191 | 0.84 (0.57, 1.24) |
|  |  |  |  |  |  |  | |  |  |  |  |  |  |
| **Living with a smoker** | 5185 | 0.89 (0.70, 1.13) | 5185 | 0.82 (0.68, 1.00) | 5185 | 0.82 (0.68, 0.99) | | 4457 | 0.79 (0.61, 1.02) | 4457 | 0.84 (0.69, 1.02) | 4457 | 0.90 (0.75, 1.09) |

^a^ Sample only includes baseline smokers and those with missing follow-up data are counted as smokers; ^b^ Sample only includes baseline non-smokers and those with missing follow-up data are counted as non-smokers; ^c^ Adjusted for baseline smoking status of resident, student gender, family affluence score (0-2, 3-4, 4-6), family vehicle ownership (no family car or van, one family car or van, two family cars or vans) and stratification variables (country: England or Wales; type of school: independent or state; mixed-sex or single-sex; English or Welsh speaking; size of school year group: < 200, ≥200; % students entitled to free school meals: ≤19%, >19%); ^d^ Unable to estimate as no cases of cessation within strata of private schooling.
